# Optimizing RetinaNet anchors using differential evolution for improved object detection

**DOI:** 10.1038/s41598-025-02888-x

**Published:** 2025-06-20

**Authors:** Asaad Mohammed, Hosny M. Ibrahim, Nagwa M. Omar

**Affiliations:** Information Technology Department, Faculty of Computers and Information, Assiut University, Assiut, 71515 Egypt

**Keywords:** RetinaNet, Anchor optimization, Differential evolution, Object detection, Deep learning, Computer vision, Computer science, Information technology

## Abstract

Object detection is a fundamental task in computer vision. It has two primary types: one-stage detectors known for their high speed and efficiency, and two-stage detectors, which offer higher accuracy but are often slower due to their complex architecture. Balancing these two aspects has been a significant challenge in the field. RetinaNet, a premier single-stage object detector, is renowned for its remarkable balance between speed and accuracy. Its success is largely due to the groundbreaking focal loss function, which adeptly addresses the issue of class imbalance prevalent in object detection tasks. This innovative approach significantly enhances detection accuracy while maintaining high speed, making RetinaNet an ideal choice for a wide range of real-world applications. However, its performance decreases when applied to datasets containing objects with unique characteristics, such as objects with elongated or squat shapes. In such cases, the default anchor parameters may not fully meet the requirements of these specialized objects. To overcome this limitation, we present an enhancement to the RetinaNet model to improve its ability to handle variations in objects across different domains. Specifically, we propose an optimization algorithm based on Differential Evolution (DE) that adjusts anchor scales and ratios while determining the most appropriate number of these parameters for each dataset based on the annotated data. Through extensive experiments on datasets spanning diverse domains such as the Karlsruhe Institute of Technology and Toyota Technological Institute (KITTI), the Unconstrained Face Detection Dataset (UFDD), the TomatoPlantFactoryDataset, and the widely used Common Objects in Context (COCO) 2017 benchmark, we demonstrate that our proposed method significantly outperforms both the original RetinaNet and anchor-free methods by a considerable margin.

## Introdction

Object detection is a pivotal task in computer vision, involving the identification and localization of objects within images or video frames. Its applications span a wide range of fields, including autonomous vehicles^1,2^, surveillance^3,4^, medical imaging^5,6^ and augmented reality^7,8^. The evolution of object detection methods has seen remarkable progress over the last decade, transitioning from traditional methods to more sophisticated deep learning-based methods. In early traditional methods, such as the Scale-Invariant Feature Transform (SIFT)^9^ and the Histogram of Oriented Gradients (HOG)^10^, extracting strong features from images was crucial. Both methods typically used sliding window techniques to detect objects, which, despite their effectiveness, were computationally expensive and lacked real-time capabilities. With the emergence of machine learning, more sophisticated techniques like the Deformable Part Models (DPM)^11^, emerged. These significantly enhanced the detection accuracy by modeling objects as a collection of parts. A significant breakthrough in object detection emerged with the advent of deep learning, particularly Convolutional Neural Networks (CNNs)^12,13^, which substantially enhanced the performance of object detectors and surpassed the capabilities of traditional methods.

**Fig. 1:**
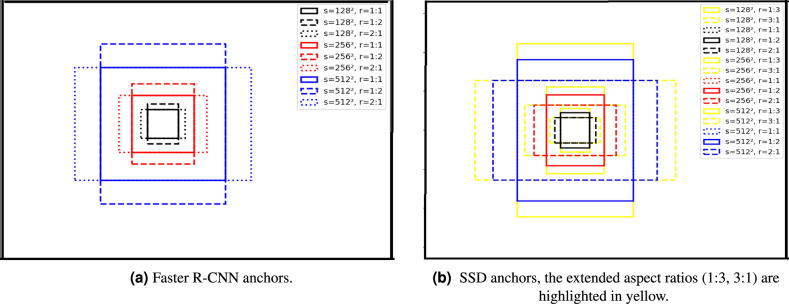
A visualization of anchor boxes of Faster R-CNN^12^ and SSD^14^.

**Fig. 2:**
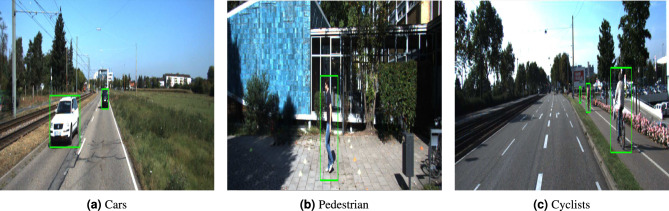
The variations in size and shape between cyclist and pedestrian compared to car. Images from KITTI dataset^18^.

A key component in many state-of-the-art object detection models^12,13^ are anchor boxes^14^, also known as default boxes^15^, which are predefined bounding boxes of various scales and ratios that propose potential locations of objects within an image for localization. Each object detector defines anchors with different scales and ratios. For example, in the two-stage method Faster R-CNN^12^, the authors use three scales with box areas of {$128^2$, $256^2$, $512^2$}, and three aspect ratios {1:1, 1:2, 2:1}, resulting in a total of 9 anchors. The one-stage method Single Shot MultiBox Detector(SSD)^14^ extends this configuration by adding two additional aspect ratios {1:3 , 3:1} to better detect objects with elongated or squat shapes. Figure 1 illustrates the default anchor boxes used in both models. Other methods, such as Cascade R-CNN^13^, RefineDet^16^, and Guided Anchoring^17^, also adopt a similar strategy and incorporate anchor boxes with standardized scales and ratios to cover a wider range of object shapes and improve accuracy. However, these default anchor boxes may not be suitable for detecting objects with specific characteristics. For example, in the KITTI dataset^18^, which is widely used in autonomous driving research, objects such as pedestrians and cyclists are often much smaller and have different shapes compared to cars. Thus, the default anchor boxes may not align well with these objects, leading to decreased detection performance. Figure 2 shows the differences in shape and size between pedestrians, cyclists, and cars. For anchor-based methods to achieve higher performance, they require careful tuning of anchor parameters to better align with object characteristics. The selection of these parameters, including the number, scales, and aspect ratios of anchors significantly impacts both detection accuracy and computational efficiency. An inappropriate selection of these parameters can hinder anchor-based methods from accurately localizing objects, resulting in lower accuracy. To address these challenges, researchers have developed anchor-free methods, which bypass the need for predefined anchor boxes by directly predicting object locations and dimensions within the image. Notable examples of such approaches include FSAF^19^ and FCOS^20^, which focus on the object’s center as the primary reference for detection, classifying them as center-based methods. In contrast, keypoint-based methods, such as CenterNet^21^ and ExtremeNet^22^, leverage self-learned keypoints to define bounding boxes, moving beyond the sole reliance on the object center. Although anchor-free methods eliminate the dependence on predefined anchor boxes, they come with certain limitations. Specifically, these methods face challenges in detecting small and occluded objects, as they rely solely on image features to predict bounding boxes. Moreover, anchor-free methods often demand greater computational resources than anchor-based methods, since they perform bounding box predictions at every spatial location within the image. This leads to increased inference time and higher computational complexity for the object detector. To overcome the challenges posed by previous methods, we propose an enhancement to the RetinaNet^23^ model by developing a novel optimization algorithm based on DE^24^ for optimizing anchor parameters. The algorithm automatically selects the most appropriate anchor scales, aspect ratios, and their numbers, addressing the limitations associated with manual tuning. To validate the effectiveness of the proposed enhancement, we conducted comprehensive experiments across diverse datasets spanning multiple domains, including KITTI^18^, UFDD^25^, TomatoPlantFactoryDataset^26^ and COCO 2017^27^.

The primary contributions can be outlined as follows:

- Developed a novel optimization algorithm based on DE that automatically selected the most suitable anchor parameters for the RetinaNet model based on annotated data.
- Conducted extensive experiments across multiple datasets from diverse domains to validate the impact of optimized anchors on detection accuracy.
- Demonstrated that optimizing anchor parameters significantly outperformed anchor-free methods, reinforcing the effectiveness of anchor-based approaches when properly tuned.

## Related work

Anchor optimization is a pivotal aspect of object detection models that rely on anchor-based methods^12,23^. In recent years, significant progress has been made through various approaches aimed at optimizing anchor boxes to enhance the performance of detection models across diverse datasets. We can categorize existing optimization methods into four categories: (1) clustering-based anchor optimization, (2) dynamic anchor generation approaches, (3) trainable variables and data-driven optimization, and (4) specialized anchor optimization approaches.

### Clustering-based anchor optimization

A foundational approach to anchor optimization utilizes clustering techniques. YOLOv3^28^ applies k-means clustering to bounding boxes in the training set to automatically determine optimal anchor sizes. Similarly, the authors in^29^ employ a clustering analysis method based on Intersection over Union (IoU), prioritizing the overlap between predicted and ground-truth boxes rather than relying solely on size and shape distribution. Another work^30^ introduces a perspective-aware methodology, which segments the image into key regions using clustering techniques and subsequently applies evolutionary algorithms to optimize anchors for each region. A notable method, Data-Driven Anchor Box Optimization^31^, enhances anchor selection in UAV-based maritime search and rescue (SAR) tasks by combining IoU-based clustering with k-means clustering. This refinement improves anchor selection for two-stage detectors such as Faster R-CNN, especially when paired with Feature Pyramid Networks (FPN). By leveraging clustering, these approaches ensure that anchor boxes better align with dataset characteristics, improving detection accuracy. A more recent example is KCFS-YOLOv5, proposed by Tian et al.^32^, which enhances object detection in satellite imagery by integrating K-means-based anchor selection with additional architectural modifications. The method combines K-means clustering to optimize anchor boxes with attention mechanisms and a Bidirectional Feature Pyramid Network (BiFPN) to improve feature fusion. However, all clustering-based methods share a major limitation, they require a predefined, fixed number of clusters. This rigidity prevents anchors from fully capturing the diversity of object sizes and shapes in the dataset, leading to suboptimal performance when applied to varying datasets. In contrast, our proposed DE-based optimization method dynamically adjusts both the number and sizes of anchors during optimization. Instead of relying on static clustering results, DE iteratively refines anchor configurations based on an adaptive evolutionary strategy, ensuring an optimal distribution of anchors that effectively captures variations in object scales and aspect ratios.

### Dynamic anchor generation approaches

Dynamic anchor generation Approaches allow models to generate and modify anchors on-the-fly. For instance, MetaAnchor^33^ utilizes residual learning in combination with a two-layer neural network to dynamically generate anchors, allowing the model to adapt to variations in object sizes and shapes. Another approach, Differentiable Anchoring^34^, introduces a parallel branch alongside the classification and bounding box regression branches, enabling the model to learn anchor box sizes dynamically rather than relying on predefined values. While these methods enhance anchor flexibility, they introduce additional hyperparameters that require careful tuning, which increases the training complexity and computational overhead. Furthermore, these methods may still struggle to generalize across different datasets without extensive manual adjustments, as they lack a mechanism to efficiently explore high-dimensional search spaces for optimal anchor configurations. Our proposed DE-based optimization overcomes these limitations by eliminating the need for additional hyperparameters and complex model modifications. Instead of relying on manual tuning or predefined network structures, DE autonomously explores the search space, allowing for a more adaptive and dataset-agnostic optimization process. This ensures that anchor configurations remain robust across different datasets without requiring extensive adjustments.

### Trainable variables optimization

Another class of anchor optimization approaches treats anchor shapes as trainable parameters within the model. For instance, the approach in^35^ optimizes anchor shapes using backpropagation, enabling models to refine anchors dynamically to fit dataset-specific characteristics. While this approach offers a high degree of flexibility, it comes with significant computational overhead, increasing both training and inference complexity. One of the primary challenges associated with trainable anchor variables is the risk of overfitting. Since the model learns anchor configurations directly from the training set, it may struggle to generalize to unseen data, especially if the dataset is small or imbalanced. Additionally, these methods require multiple backpropagation steps and careful hyperparameter tuning, leading to longer convergence times and increased training instability. Our proposed DE-based optimization method directly addresses these challenges by optimizing anchor configurations externally rather than embedding them within the model itself. This eliminates the need for additional learnable parameters, reducing the risk of overfitting while maintaining adaptability across different datasets. Unlike trainable variable-based methods, DE efficiently explores the search space and optimizes anchor configurations without requiring backpropagation, making it computationally more efficient and stable.

### Specialized anchor optimization approaches

Several specialized anchor optimization strategies have been introduced to address specific object detection challenges, including small object detection, depth-based adaptation, semantic-driven anchor refinement, and metaheuristic-based optimization.

*Small Object Detection:* One approach^36^ employs the Crow Search Algorithm (CSA) to optimize anchor ratios and scales, specifically enhancing small object detection in high-resolution aerial imagery. While this method improves detection performance for small targets, its reliance on predefined scales and aspect ratios makes it less adaptable to datasets with diverse object distributions. *Depth-Based Anchor Optimization:* Another method^37^ leverages depth information to dynamically estimate anchor sizes based on object distances within a scene. This ensures that anchor boxes better correspond to object sizes in 3D space. However, this approach is highly dependent on accurate depth estimation, making it vulnerable to errors when dealing with noisy or incomplete depth data. *Semantic-Guided Anchor Adaptation:* Semantic cues have also been used to refine anchor placement, particularly in specialized domains such as Synthetic Aperture Radar (SAR) imagery^38^. While this method improves detection performance in structured environments, it is highly sensitive to segmentation errors, which can lead to suboptimal anchor configurations and degraded performance. *Metaheuristic-Based Optimization:* Recent works have explored the use of metaheuristic algorithms to optimize anchor related parameters and model configurations, particularly in domain-specific contexts like remote sensing. For example, Elgamily et al.^39^ applied a suite of metaheuristic and hybrid metaheuristic optimizers including Genetic Algorithms (GA), Particle Swarm Optimization (PSO), and GA-PSO hybrids to tune hyperparameters of YOLOv7^40^ and YOLOv8^41^ for object detection in satellite imagery. Their results demonstrated notable improvements in detection accuracy and robustness, especially for small and densely packed objects. However, their method primarily focused on tuning general model-level hyperparameters, not anchor configurations directly, which can limit adaptability across different datasets. Our proposed method overcomes these limitations by dynamically evolving anchor configurations without relying on fixed scales, depth information, or semantic segmentation. Unlike depth-based or semantic-guided techniques, DE operates directly on object distributions within the dataset, making it a more robust and adaptable solution for diverse object detection tasks. Moreover, in contrast to metaheuristic-based methods, which primarily focus on tuning general model-level hyperparameters, the proposed method specifically targets anchor box optimization. This allows it to achieve more precise localization and scale adaptation, especially in datasets with high object variability, without requiring architecture-specific adjustments. A detailed comparison of previous methods, including their strengths and weaknesses, is provided in Table 1.

## Proposed work

We begin this section by providing a comprehensive overview of the RetinaNet^23^ architecture, which forms the backbone of our approach. Subsequently, we detail the proposed algorithm for optimizing RetinaNet’s anchor parameters.

### RetinaNet model

**Fig. 3:**
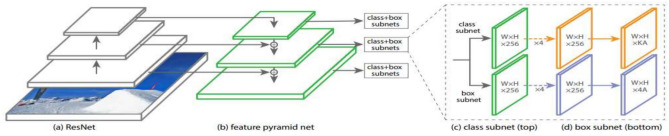
RetinaNet model architecture, as depicted in^23^.

RetinaNet^23^ is a state-of-the-art one-stage object detection model. It addresses the common class imbalance problem in object detection tasks through a novel focal loss function. This loss function down weights the loss assigned to well-classified examples, and focusing more on hard examples, which significantly improves detection performance. The architecture of the model is built upon a ResNet backbone network^42^ combined with a Feature Pyramid Network (FPN)^43^. The former network is responsible for extracting high-level features from the input images, then proceed these features to FPN network^43^. The FPN network^43^ then enhances the standard feature hierarchy of the backbone network with lateral connections and top-down pathways, creating a rich multi-scale feature pyramid. Which allows RetinaNet^23^ to detect objects at multiple scales effectively. The model also employs two subnetworks, a classification subnet, that predicts the probability of object presence at each spatial position, and a bounding box regression subnet, that refines the anchor boxes to better fit the detected objects. Figure 3 provides visual illustration of the RetinaNet architecture. Due to its innovative design, RetinaNet combines the speed advantage of one-stage detectors while maintaining the accuracy associated with two-stage detectors, making it highly efficient for real-world applications. So, we selected RetinaNet^23^ as the backbone of our work.

**Table 1:** Comparison of various object detection methods with anchor optimization techniques.

Category	Method	Strengths	Weaknesses
Clustering-based optimization	YOLOv3^28^	Automatically determines anchor sizes Effective for datasets with well-defined objects	Requires a fixed number of clusters May not generalize to diverse object distributions
	Data-Driven Anchor Optimization^31^	Differential es anchors based on dataset characteristics Effective in specialized applications like UAV SAR	Requires large labeled datasets Increases training instability due to backpropagation
	Faster R-CNN+FP+GN+K-means^29^	Focuses on overlap rather than just size Can improve anchor-object alignment	Still requires a predefined cluster count Limited adaptability to varied datasets
	KCFS-YOLOv5^32^	Combines K-means anchor optimization with attention mechanisms and BiFPN Improves detection accuracy in satellite imagery	Relies on fixed cluster count Architecture-specific; limited generalization
Dynamic anchor generation	MetaAnchor^33^	Dynamically generates anchors Adapts to variations in object sizes	Introduces additional hyperparameters Increases training complexity
	Differentiable Anchoring^34^	Learns optimal anchor sizes automatically Enhances detection without manual tuning	Adds computational overhead May struggle to generalize across datasets
Trainable variables optimization	Opt (k-means)^35^	Learns anchor configurations directly from data Enhances accuracy in domain-specific applications	Risk of overfitting to training data Adds computational overhead
Specialized anchor optimization	RetinaNet with Anchor Optimization (Small Object Detection)^36^	Optimizes anchor scales and ratios Effective for small object detection	Uses fixed scales/ratios, limiting adaptability May not generalize to different datasets
	Depth-based Anchor Optimization^37^	Adjusts anchor sizes dynamically using depth Enhances scene-specific detection	Relies on accurate depth information Sensitive to noise in depth measurements
	HTC+^38^	Uses semantic cues for anchor refinement Improves performance in structured environments	Dependent on accurate semantic segmentation Errors in segmentation lead to suboptimal anchors
	YOLOv7/YOLOv8 + GA/PSO Optimization^39^	Applies Genetic Algorithms, PSO, and hybrid metaheuristics to improve detection in satellite imagery Boosts accuracy, especially for small and dense objects	Focuses on global hyperparameter tuning, not anchor-specific optimization Requires careful tuning of optimizer parameters

### The proposed RetinaNet anchors optimization

The process of anchor generation in RetinaNet^23^ involves creating anchors with areas ranging from $32^2$ to $512^2$ across pyramid levels $P_3$ to $P_7$. At each level, three aspect ratios {1:2, 1:1, 2:1} and three scales { $2^{0/3}$, $2^{1/3}$, $2^{2/3}$} are used to create a diverse set of anchors. This configuration allows the model to detect objects of various sizes and shapes. Unfortunately, these default anchor parameters may not be optimal for all datasets, particularly for objects with unique shapes and aspect ratios^18,25,44^. To address this limitation, we introduce a DE^24^-based optimization algorithm that systematically tunes anchor scales, aspect ratios, and the total number of anchors for improved detection performance. DE is a population-based optimization algorithm designed to optimize both continuous and discrete variables, making it well-suited for selecting the most effective anchor parameters in RetinaNet^23^. Unlike traditional gradient-based methods, DE does not require derivative information and can efficiently explore high-dimensional search spaces while avoiding local minima. The optimization process consists of two main phases: **Initialization** and **Evolution**.

*Initialization Phase:* In this phase, a population of candidate solutions at generation *G* $(G \in \{1, \ldots ,\textbf{G}_{max} \})$ is $\textbf{P}^G = \{X_j^G : j = 1, 2, \ldots , N\}$  where *N* denotes the size of population. Each candidate solution $X_j^G$ in the population is a vector of variable size, $X_j^G = \{ x_{0,j}^G, x_{1,j}^G, \ldots , x_{D-1,j}^G \}$, where *D* represents the size of the vector. These vectors define a potential set of scales, aspect ratios, and their respective counts. Each element $X^G_{z,j}$ within the vector is initialized using a randomized approach as described in Eq. (1):

1 $$\begin{aligned} X_{z,j}^G = X_{\text {low},z} + (X_{\text {upp},z} - X_{\text {low},z}) * \text {rand}(0, 1) \end{aligned}$$

where $z$ indexes the element in the vector $z \in \{0, 1, \ldots , D-1\}$, and $X_{\text {low,z}}$, $X_{\text {upp,z}}$ represent the lower and upper bounds search space of the *z*-th element, respectively. For our specific case, the boundary values for scales and aspect ratios are derived from the bounding box coordinates in the annotated dataset. Let $k$ be the number of anchor boxes in the dataset. The $i$- th anchor box, where $i \in \{0, 1, \ldots , k-1\}$, is denoted as $K_i$, and defined by its corner coordinates $(x_{1,i},y_{1,i},x_{2,i},y_{2,i})\in \mathbb {R}^{4}$, where $(x_{1,i},y_{1,i})\ and\ (x_{2,i},y_{2,i})$ denote the top-left and bottom-right corners, respectively. The scale $S_i$ of an anchor $K_i$ is computed as the ratio of the anchor area to the image size, as shown in Eq. (2):

2 $$\begin{aligned} S_i = \sqrt{\frac{(x_{2,i} - x_{1,i}) \times (y_{2,i} - y_{1,i})}{W \times H}} \end{aligned}$$

where *W* and *H* represent the image width and height, respectively. To determine the minimum and maximum scale boundaries for optimization, we use the following equations:

3 $$\begin{aligned} & \text {S}_{\min } = \min _{0 \le i < k} \left( \sqrt{\frac{(x_{2,i} - x_{1,i}) \times (y_{2,i} - y_{1,i})}{W \times H}}\ \right) \end{aligned}$$

4 $$\begin{aligned} & \text {S}_{\max } = \max _{0 \le i < k} \left( \sqrt{\frac{(x_{2,i} - x_{1,i}) \times (y_{2,i} - y_{1,i})}{W \times H}}\ \right) \end{aligned}$$

Similarly, the aspect ratio $R_i$ of an anchor $K_i$ is defined as the ratio of its height to width, as given in Eq. (5):

5 $$\begin{aligned} \text {R}_{i} =\left( \frac{y_{2,i} - y_{1,i}}{x_{2,i} - x_{1,i}}\right) \end{aligned}$$

The minimum and maximum aspect ratio boundaries are computed as:

6 $$\begin{aligned} & \text {R}_{\min } = \min _{0 \le i < k} \left( \frac{y_{2,i} - y_{1,i}}{x_{2,i} - x_{1,i}}\right) \end{aligned}$$

7 $$\begin{aligned} & \text {R}_{\max } = \max _{0 \le i < k} \left( \frac{y_{2,i} - y_{1,i}}{x_{2,i} - x_{1,i}}\right) \end{aligned}$$

Lastly, the number of scales and aspect ratios is selected manually based on dataset characteristics.

*Evolution Phase:* In this phase, the algorithm performs three key operations: **mutation**, **crossover**, and **selection**.

*Mutation*: A mutant vector $V_{j}^{G}$ is generated for each target vector $X_{j}^{G}$ at generation $G$, as shown in Eq. (8):

8 $$\begin{aligned} V_{j}^{G} = X_{a}^{G} + F * (X_{b}^{G} - X_{c}^{G}) \end{aligned}$$

where, $F$ is the scaling factor (typically in the range [0,1]), and $X_a^G, X_b^G,$ and $X_c^G$ are randomly chosen vectors from the population, ensuring $a \ne b \ne c \ne j.$.

*Crossover:* The crossover operation is performed between the target vector $X_{j}^{G} = \{x_{1,j}^{G}, x_{2,j}^{G}, \ldots , x_{D,j}^{G}\}$ and the mutant vector $V_{j}^{G} = \{v_{1,j}^{G}, v_{2,j}^{G}, \ldots , v_{D,j}^{G}\}$ to create a trial vector $U_{j}^{G}$. governed by a crossover probability $C_r$, as described in Eq. (9):

9 $$\begin{aligned} u_{m,j}^{G} = {\left\{ \begin{array}{ll} v_{m,j}^{G} & \text {if } \text {rand}_j \le C_r \\ x_{m,j}^{G} & \text {otherwise} \end{array}\right. } \end{aligned}$$

where $$m \in \{1, 2, \ldots , D\}$$ and $$C_r \in [0, 1]$$.

*Selection:* The final step selects the best solutions among the trial and target vectors. If a trial vector achieves a better objective function score, it replaces the corresponding target vector in the next generation. If not, the target vector is carried forward to the next generation. The objective function is designed to maximize *IoU* score between a predicted bounding box, $\textbf{B}_{\text {pred}}$ and a ground truth bounding box, $\textbf{B}_{\text {gt}}$, as defined in Eq. (10)^45^:

10 $$\begin{aligned} IoU = \frac{\text {Area}(\textbf{B}_{\text {pred}} \cap \textbf{B}_{\text {gt}})}{\text {Area}(\textbf{B}_{\text {pred}} \cup \textbf{B}_{\text {gt}})} \end{aligned}$$

A higher *IoU* value indicates better alignment between the predicted and actual object locations. The evolution phase continues until the stopping criterion is met, either achieving the desired *IoU* threshold or reaching the maximum number of generations.

Compared to traditional optimization techniques such as Bayesian optimization^46^, genetic algorithms^47^, and grid search^48^, DE^24^ is computationally efficient, self-adaptive, and highly effective at finding globally optimal solutions without requiring extensive hyperparameter tuning. Grid Search, despite its simplicity, suffers from an exponential increase in computational cost as the number of parameters grows, making it impractical for high-dimensional search spaces. Bayesian optimization, while efficient in continuous spaces, struggles with discrete variables and demands significant computational resources to model the search space probabilistically. Genetic algorithms, though capable of avoiding local minima, require careful tuning of crossover and mutation rates, leading to increased complexity. In contrast, DE balances exploration and exploitation, allowing for efficient optimization with minimal hyperparameter dependencies. The results demonstrate that DE-based anchor optimization significantly improves object detection accuracy, outperforming both manually tuned anchor-based methods and anchor-free approaches. A detailed comparison of these optimization techniques is provided in Table 2, emphasizing the advantages of DE in terms of scalability, adaptability, and computational efficiency. The pseudocode of DE is presented in Algorithm 1.

**Algorithm 1:**
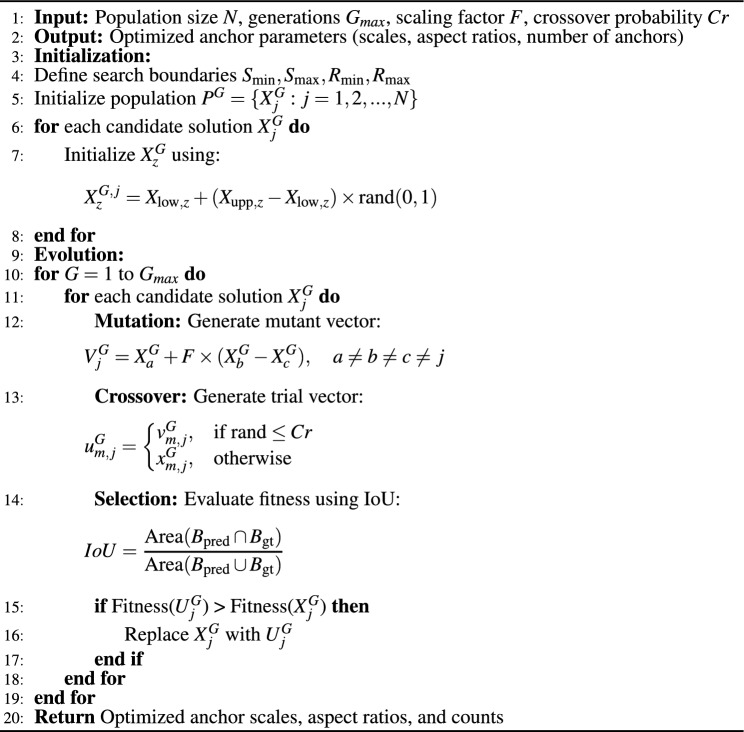
Differential evolution-based anchor optimization for RetinaNet

**Table 2:** Comparison of different optimization techniques.

Optimization method	Scalability	Handles discrete & continuous	Avoids local minima	Computational cost	Hyperparameter tuning
Grid search^48^	Low	$\times$	$\times$	High	Extensive
Bayesian algorithm^46^	Moderate	$\times$	$\checkmark$	Very high	Medium
Genetic algorithm^47^	High	$\checkmark$	$\checkmark$	High	High
Differential evolution (DE)^24^	High	$\checkmark$	$\checkmark$	Moderate	Low

## Experiments

In this section, we first provide an overview of each dataset used in our experiments. Then, we describe the experimental setup and implementation details of both the RetinNet^23^ and our proposed optimization algorithm. Finally, we present and discuss the results obtained on each dataset, and the performance of the Optimized RetinaNet (OptRetinaNet) is evaluated against the original RetinaNet^23^ and a diverse set of recent object detectors, including anchor-free methods^19,20^, transformer-based architectures^49,50^, and recent unsupervised methods^51,52^.

### Datasets

**Fig. 4:**
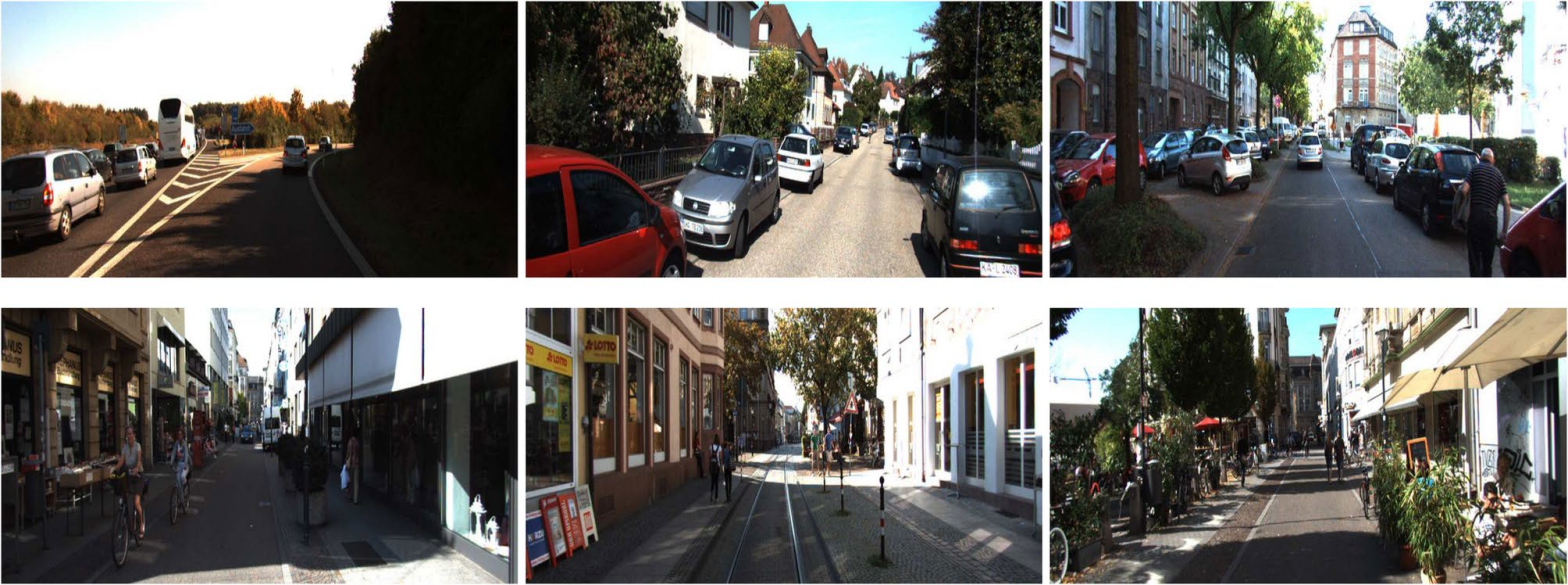
A sample of images from the KITTI dataset, showcasing different object categories and environmental conditions.

*KITTI*^18^ *dataset* : is a widely used in autonomous driving research. It provides 7481 images for training and 7518 images for testing, each accompanied by camera calibration files to ensure accurate spatial representation. It includes annotations for various object classes such as cars, pedestrians, cyclists and other objects, which makes it a comprehensive resource for training and evaluating object detection models in complex urban environments.A sample of images from the KITTI dataset, is presented in Fig. 4.

**Fig. 5:**
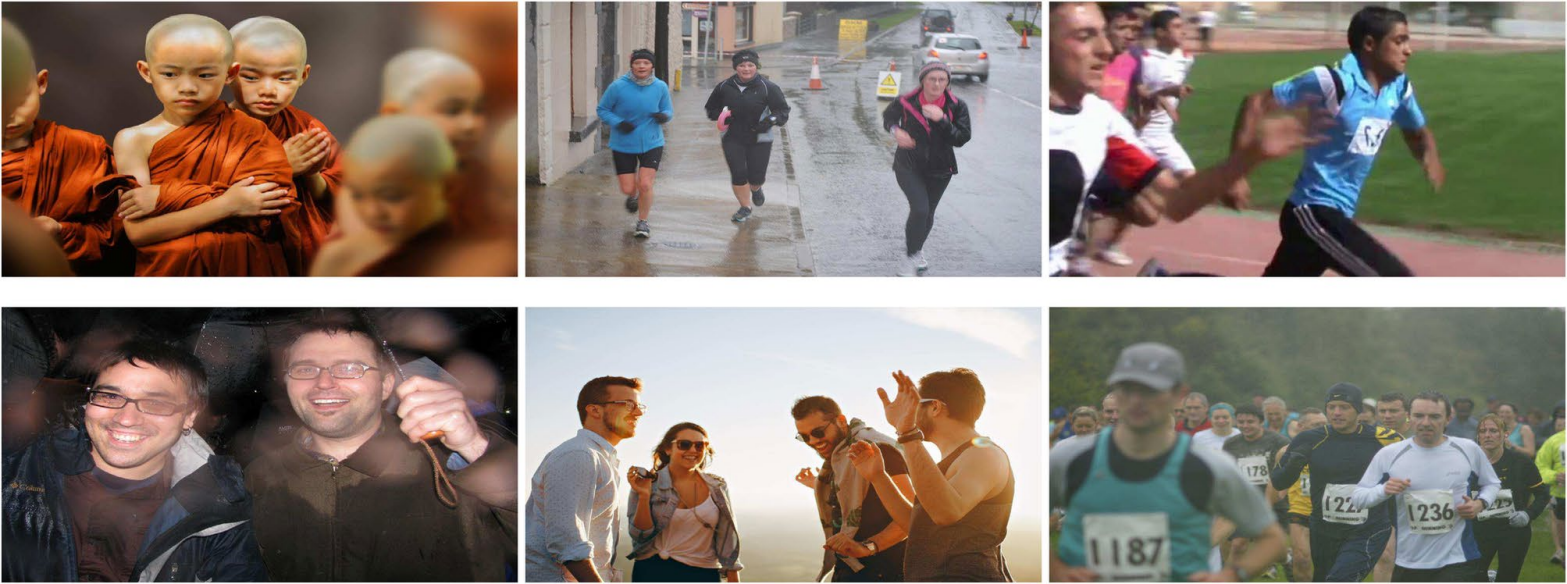
A sample of images from the UFFD dataset, showcasing various face detection challenges, including motion blur, lens impediments, haze, rain, and illumination variations.

*UFFD*^25^ *dataset* : is a challenging dataset specifically designed to evaluate face detection models under various adverse conditions. It comprises 6424 images with 10,895 face annotations, capturing a wide range of real-world environmental weather conditions and other degradations such as lens impediments, motion blur and focus blur. Additionally, it includes many distractor images containing non-human faces such as animal face or no faces at all, which are crucial for measuring the performance of face detectors in rejecting non-face images. A sample of images from the UFDD dataset, is presented in Fig. 5.

**Fig. 6:**
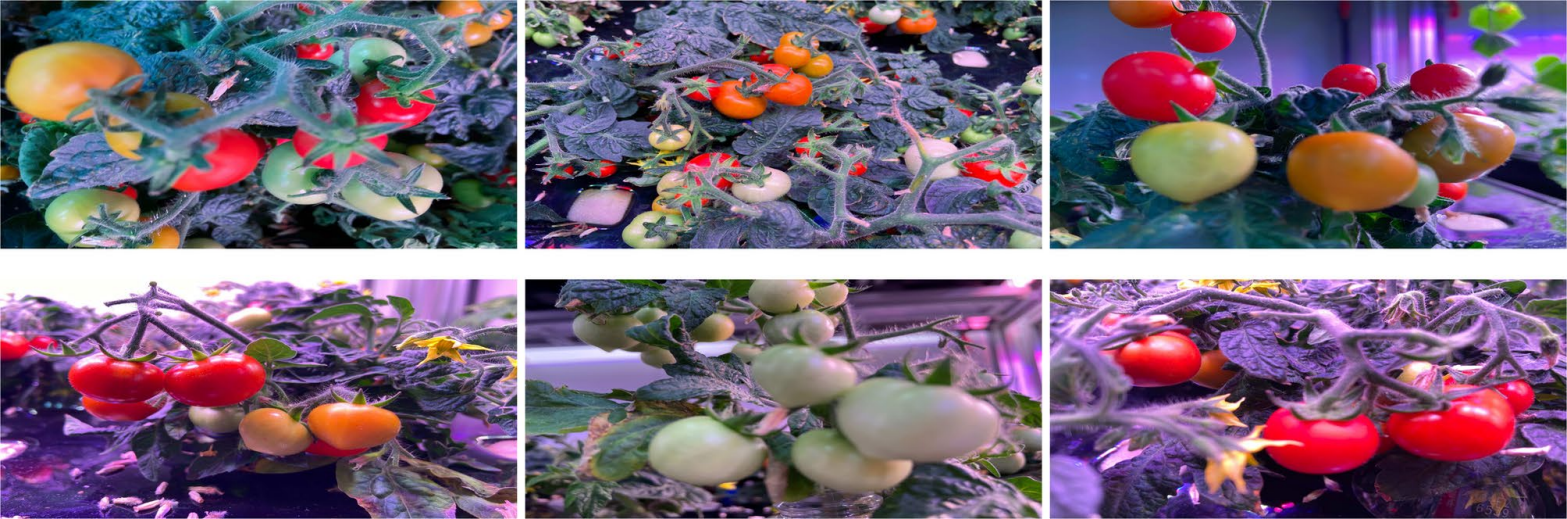
A sample of images from the TomatoPlantFactory dataset, showcasing various challenges in tomato detection, including complex ambient lighting, occlusions and background clutter.

*TomatoPlantFactoryDataset*^26^: is a comprehensive collection of high quality images, which is designed to facilitate advanced research in tomato plant detection. It comprises 520 images with 9112 tomato fruit instances classified as red and green tomatoes. Unlike existing datasets, which typically feature lower-quality images at around 1 MP (1270 $\times$ 720) due to poor sensor performance, this dataset offers significantly higher imaging quality and more pixel information. Furthermore, it is enriched by the presence of complex ambient lighting, which poses additional challenges for tomato object detection, and making the dataset particularly valuable for developing and testing robust detection algorithms. A sample of images from the TomatoPlantFactoryDataset, is presented in Fig. 6.

**Fig. 7:**
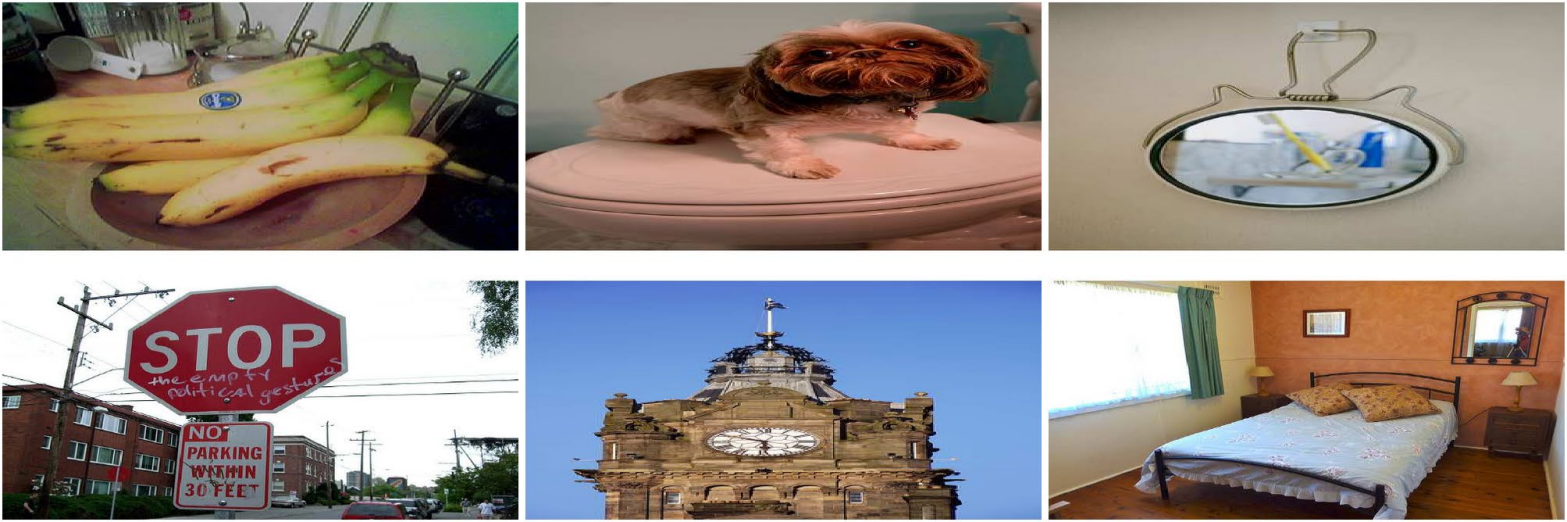
A sample images from the MS COCO 2017 dataset, showcasing diverse object categories and real-world scene complexity.

*MS COCO 2017*^27^ *dataset*: The Microsoft Common Objects in Context (MS COCO) 2017 dataset is a widely recognized benchmark in the field of object detection, segmentation, and image captioning. It comprises approximately 118,000 training images and 5,000 validation images, with over 2.5 million annotated object instances spanning 80 everyday object categories. Each image typically contains multiple objects at different scales and orientations, often appearing in non-iconic views and within cluttered scenes characteristics that make COCO particularly challenging and realistic. A sample of images from the MS COCO 2017, is presented in Fig. 7. A summary of the datasets used, including the number of images, annotations, object classes, key challenges, and domain, is presented in Table 3.

### Experimental setup and implementation details

#### Computational environment

All experiments were conducted on a high-performance machine equipped with an NVIDIA GeForce GTX 1080 Ti GPU, 32 GB of RAM, and an Intel Core i7 @ 3.40GHz CPU. The implementation was carried out using the MMDetection framework ^53^.

#### Implementation parameters

For the proposed differential optimization algorithm, the population size was set to 30, and the algorithm was run for 100 generations. The scaling factor $F$ was set to 0.5, and the crossover probability $Cr$ was set to 0.9. For anchor parameters, we adopted the same configurations for base sizes, strides, and pyramid levels as those employed in the original RetinaNet ^23^. For training, we utilized pre-trained weights from the ImageNet ^54^ dataset to initialize the network. We employed mini-batch stochastic gradient descent (SGD) as the optimizer, with momentum set to 0.9 and weight decay set to 0.0001. The model was trained with an initial learning rate of 0.01 for the first 120,000 iterations, after which the learning rate was reduced by a factor of 10 every 40,000 iterations, continuing for a total of 240,000 iterations. To enhance generalization, data augmentation techniques were applied, including image resizing to 1333$\times$800 while preserving aspect ratio, random horizontal flipping $p=0.5$, color jitter with brightness, contrast, and saturation adjustments in the range of (0.8, 1.2), and scaling variations between 0.8 and 1.2. Padding with a size divisor of 32 ensured compatibility with FPN, while multi-scale testing without flipping improved robustness across object sizes. This configuration optimized anchor adaptability and detection performance while maintaining computational efficiency.

**Table 3:** Summary of the datasets used in this study, including image counts, annotations, object classes, key challenges, and domains.

Dataset	Total images	Annotations	Object classes	Key challenges	Domain
KITTI^18^	7481 (train) 7518 (test)	80.256	Cars, pedestrians, cyclists, others	Occlusions, varying lighting, perspective distortion	Autonomous driving
UFFD^25^	6424	10,895	Human faces, non-human faces, no-face images	Motion blur, lens impediments, non-human distractors	Face detection
TomatoPlantFactory^26^	520	9112	Red tomatoes, green tomatoes	Complex ambient lighting, high object density	Agricultural object detection
MS COCO 2017^27^	118K (train) 5000 (val)	886,284	80 common objects (e.g., person, car, dog, etc.)	Small object detection, cluttered scenes, high inter-class variation	General-purpose object detection

### Evaluation metrics

To assess the performance of proposed method, we use Average Precision (AP)^45^, a widely adopted metric for evaluating detection accuracy. The IoU threshold defines when a predicted bounding box is considered a valid detection, as formulated in Eq. (10). AP denotes the average precision value of the model under each recall value, representing the area under the Precision-Recall (P-R) curve. It provides a comprehensive measure of detection accuracy for a given category, balancing both precision and recall. The *AP* metric is computed as:

11 $$\begin{aligned} AP = \int _0^1 P(r) dr \end{aligned}$$

where is the precision as a function of recall. For the KITTI^18^ dataset, we follow standard evaluation settings by using an *IoU* threshold of 0.7 for cars and 0.5 for pedestrians and cyclists. These thresholds align with KITTI benchmark requirements, ensuring fair comparisons. For the UFFD^25^ dataset, we use an *IoU* threshold of 0.5, following prior face detection studies. Similarly, for the TomatoPlantFactoryDataset^26^, an *IoU* threshold of 0.5 is applied, consistent with agricultural object detection benchmarks^55^. For the MS COCO 2017^27^ dataset, we adopt the official COCO style evaluation, reporting *AP*, $AP_{50}$, $AP_{75}$, $AP_{S}$, $AP_{M}$, and $AP_{L}$, which capture average precision at multiple *IoU* thresholds and across object sizes (small, medium, large). These metrics provide a standardized and comprehensive view of detection performance on a challenging and diverse benchmark.

### Results and discussion

**Fig. 8:**
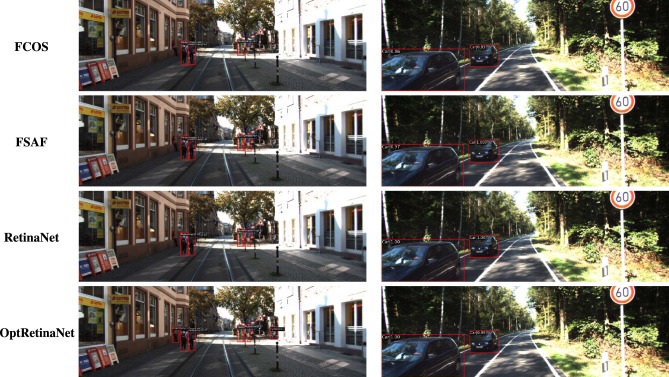
Some detection results from the four models on the KITTI^18^ dataset.

**Fig. 9:**
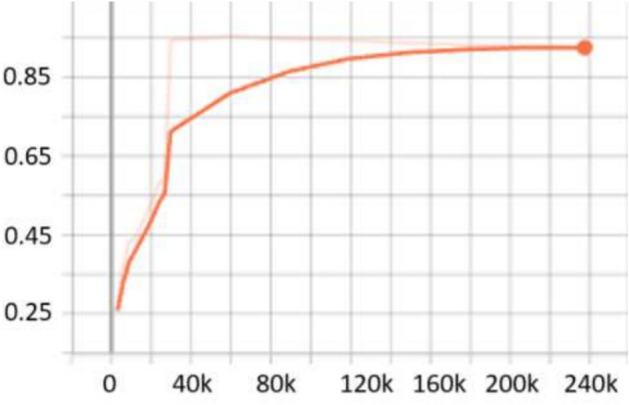
AP performance across epochs, illustrating improvements in detection accuracy over time.

**Fig. 10:**
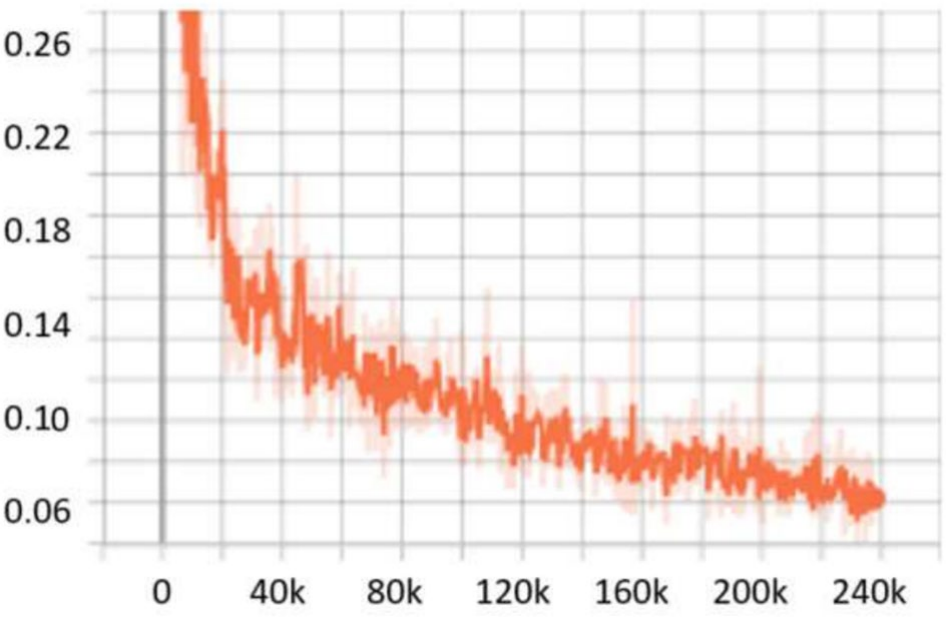
Training loss over epochs, demonstrating model convergence and stability.

For the **KITTI**^18^ dataset, the scale search boundary was set to [0.001, 0.52], where 0.001 represents the minimum scale as shown in Eq. (3) and 0.52 represents the maximum scale as shown in Eq. (4). Similarly, the aspect ratio search boundary was set to [0.25, 10], with 0.25 corresponding to the minimum ratio as shown in Eq. (6) and 10 corresponding to the maximum ratio as shown in Eq. (7). The number of scales and aspect ratios was determined through multiple experimental iterations, and it was found that using a range of 3–10 values effectively captures the diversity of object sizes while balancing detection accuracy and model complexity. The final optimized anchor configuration consisted of five aspect ratios [0.25, 0.45, 1.0, 2.15, 2.85] and three scales [0.3, 0.4, 0.5]. Since the ground truth annotations for the testing dataset are not available, the training set was split into a 3:1 ratio for training and validation. The evaluation metric used was *AP*, a standard metric for object detection. *IoU* thresholds were set to 0.7 for cars and 0.5 for pedestrians and cyclists, following KITTI^18^ dataset benchmarking standards. The results show that OptRetinaNet outperforms all evaluated models, achieving the highest *AP* scores for both ResNet-50 and ResNet-101 backbones. With ResNet-50, OptRetinaNet reached 90.3% AP for cars, 84.2% for pedestrians, and 91.0% for cyclists, significantly improving over the baseline RetinaNet, which achieved 89.6%, 80.8%, and 86.5%, respectively. The anchor-free methods FCOS and FSAF performed worse, especially in pedestrian and cyclist detection, where OptRetinaNet provided a 3.4% and 4.5% boost, respectively. DETR, while competitive, achieved only 90.1%, 82.6%, and 88.8%, falling short of the optimized RetinaNet configuration. When switching to ResNet-101, OptRetinaNet achieved even greater accuracy, reaching 93.2% for cars, 84.5% for pedestrians, and 90.6% for cyclists, outperforming all other models, including DETR, which obtained 91.7%, 83.9%, and 90.2%. The deeper ResNet-101 backbone provided an overall boost in AP across all models, confirming that a stronger feature extractor enhances object detection, but OptRetinaNet benefited the most, demonstrating the robustness of the optimized anchor configuration. Additionally, transformer-based models like Swin Transformer (Swin-T) and YOLOv8 performed worse, with Swin-T achieving only 84.5%, 74.6%, and 82.4% across the three classes, and YOLOv8 scoring 87.4%, 71.8%, and 81.0%, indicating that CNN-based architectures remain superior for the KITTI dataset. The results demonstrate that OptRetinaNet’s anchor optimization strategy effectively improves detection across various object categories, particularly benefiting pedestrian and cyclist detection, which are challenging for standard RetinaNet and anchor-free approaches. The detailed results are summarized in Table 4, while Fig. 8 visualizes detection examples. Additionally, Fig. 9 illustrates AP performance over epochs, showing how detection accuracy steadily improves over time, while Fig. 10 presents training loss curve, highlighting the model’s stability and convergence.

**Table 4:** The detailed results for object detection on the KITTI^18^ are presented in terms of AP.

Model	Backbone	Car (%)	Pedestrian (%)	Cyclist (%)
FCOS^20^	ResNet50	87.4	80.2	86.7
FSAF^19^		87.9	81.8	86.0
DETR^49^		90.1	82.6	88.8
RetinaNet^23^		89.6	80.8	86.5
OptRetinaNet		**90.3**	**84.2**	**91.0**
FCOS^20^	ResNet101	88.7	81.6	88.2
FSAF^19^		89.3	83.4	87.6
DETR^49^		91.7	83.9	90.22
RetinaNet^23^		92.9	75.8	88.9
OptRetinaNet		**93.2**	**84.5**	**90.6**
Swin-T^50^	Swin transformer	84.5	74.6	82.4
YOLOv8^41^	EfficientNet-B4	87.4	71.8	81.0

Significant values are in bold.

**Fig. 11:**
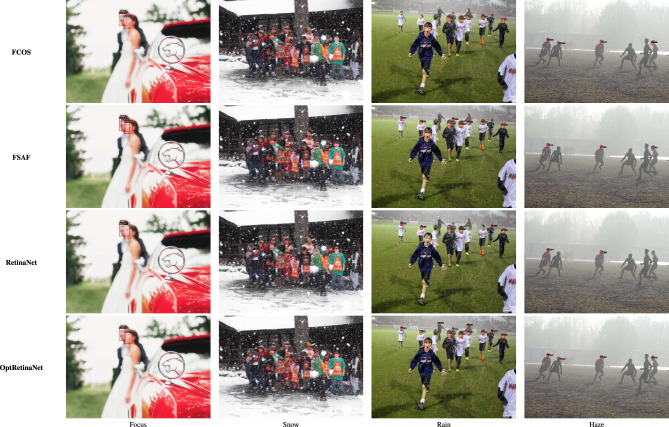
Some detection results from the four models on the UFDD^25^ dataset in different weather condition and degradation.

**Fig. 12:**
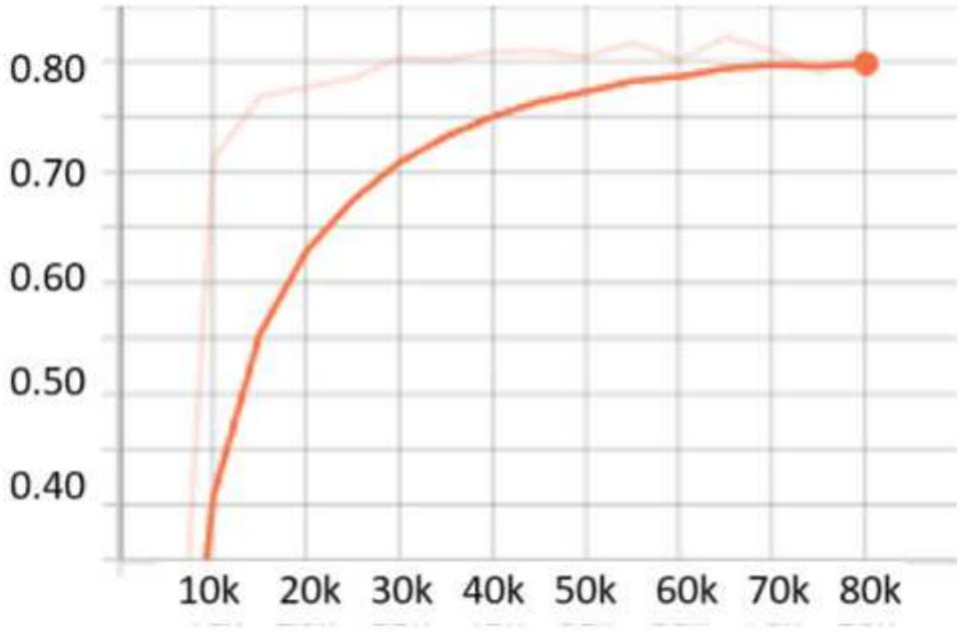
AP performance across epochs, illustrating improvements in detection accuracy over time.

**Fig. 13:**
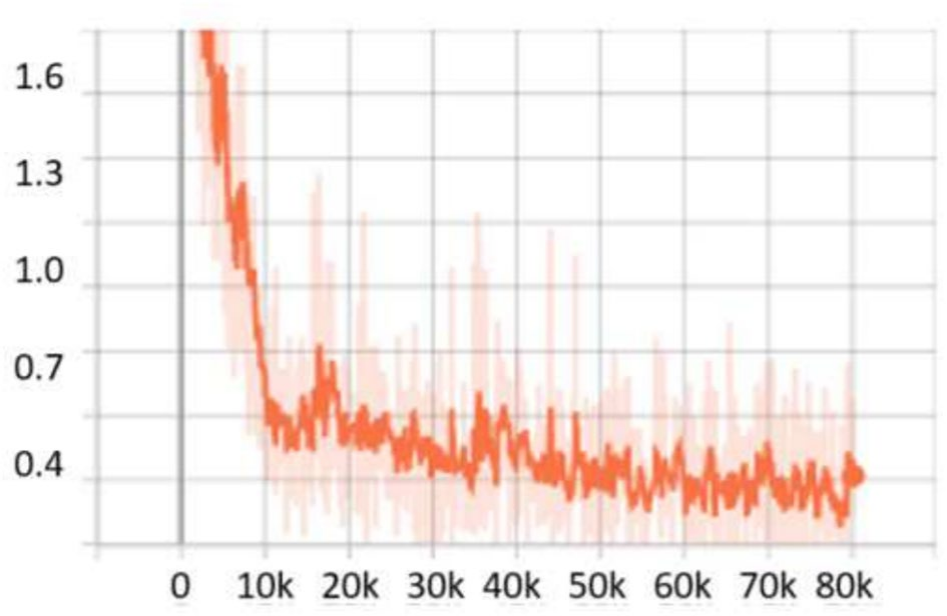
Training loss over epochs, demonstrating model convergence and stability.

For the **UFDD**^25^ dataset, the scale search boundary was set to [0.003, 0.94], where 0.003 represents the minimum scale as shown in Eq. (3) and 0.94 represents the maximum scale as shown in Eq. (4). Similarly, the boundary search for aspect ratios was set to [0.21, 6.9], where 0.21 corresponds to the minimum ratio as shown in Eq. (6) and 6.9 corresponds to the maximum ratio as shown in Eq. (7). The search boundary for the number of detected values was established through a series of experimental iterations. It was determined that a range of 5 to 15 optimally captures the diversity of face objects in the dataset. The resulting optimal values were four aspect ratios [0.2, 0.6, 1.0, 1.5] and two scales [0.2, 0.5]. The dataset was partitioned into 1,781 images for training, 296 images for validation, and 892 images for testing. For evaluation, we applied a 0.5 *IoU* threshold. Compared to RetinaNet, which achieved *AP* scores of 76.2% and 80.0% when using ResNet50 and ResNet101, respectively, OptRetinaNet exhibited significant improvements, reaching 79.7% with ResNet50 and 81.8% with ResNet101. This represents a 3.5% increase over RetinaNet when using ResNet50 and a 1.8% increase with ResNet101, demonstrating the effectiveness of the optimized anchor parameters in capturing the diverse and complex facial shapes in the UFDD dataset. Furthermore, OptRetinaNet consistently outperformed other models, including FCOS, FSAF, and DETR across both backbones. Notably, when utilizing ResNet101, OptRetinaNet achieved the highest performance, surpassing DETR (80.7%), FCOS (81.2%), and FSAF (80.3%), confirming its robustness under challenging face detection conditions such as occlusions, lighting variations, and motion blur. The detailed results are summarized in Table 5, emphasizing how OptRetinaNet consistently outperforms other anchor-based and anchor-free models. Figure 11 presents the visualized detection results from different models on the UFDD dataset, further demonstrating the superiority of OptRetinaNet in handling real-world face detection challenges under various degradations, such as haze, rain, and motion blur. Additionally, Figs. 12 and 13 illustrate the AP performance and training loss curve, respectively. Figure 12 shows how detection accuracy improves over time, highlighting the effectiveness of our optimization in refining anchor configurations and increasing AP scores across epochs. Figure 13 presents the training loss over epochs, confirming that our method maintains stable convergence and improved model generalization.

**Table 5:** The detailed results for object detection on the UFDD^25^ dataset are presented in terms of AP.

Model	Backbone	AP (%)
FCOS^20^	ResNet50	79.4
FSAF^19^		78.8
DETR^49^		79.3
RetinaNet^23^		76.2
OptRetinaNet		**79.7**
FCOS^20^	ResNet101	81.2
FSAF^19^		80.3
DETR^49^		80.7
RetinaNet^23^		80.0
OptRetinaNet		**81.8**
Swin-T^50^	Swin transformer	73.9
YOLOv8^41^	EfficientNet-B4	78.9

Significant values are in bold.

**Fig. 14:**
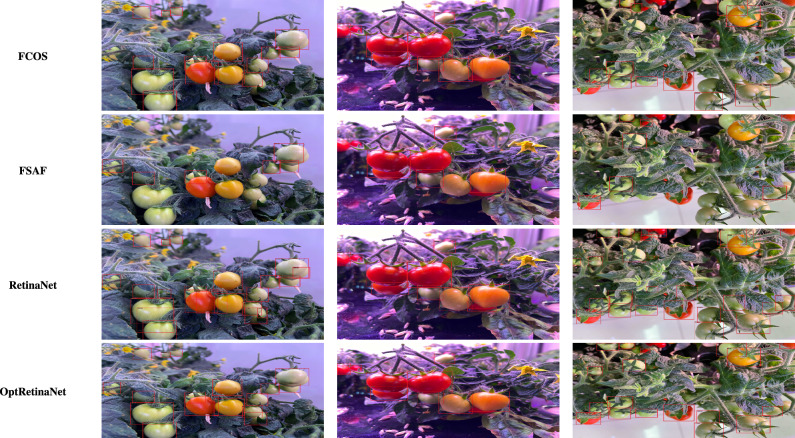
Some detection results from the models on the TomatoPlantFactoryDataset^26^.

**Fig. 15:**
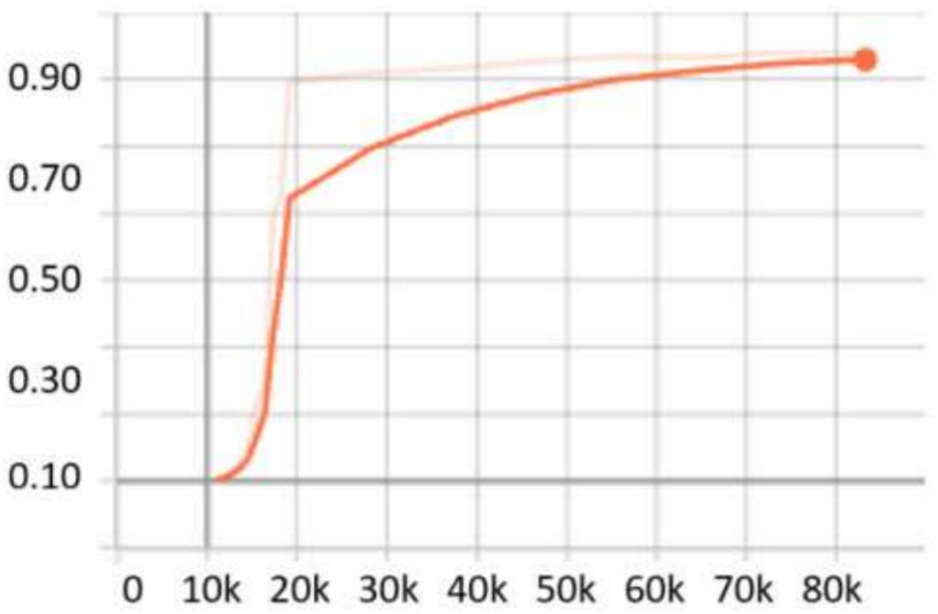
AP performance across epochs, illustrating improvements in detection accuracy over time.

**Fig. 16:**
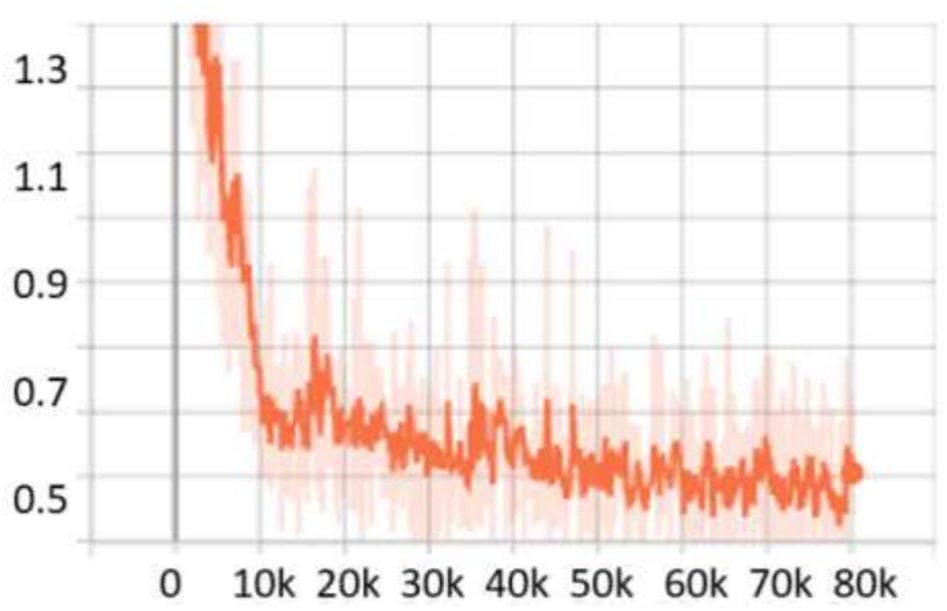
Training loss over epochs, demonstrating model convergence and stability.

For the TomatoPlantFactoryDataset^26^, the scale search boundary was set to [0.001, 0.84], where 0.001 represents the minimum scale as shown in Eq. (3) and 0.84 represents the maximum scale as shown in Eq. (4). Similarly, the boundary search for aspect ratios was set to [0.16, 5.7], where 0.16 corresponds to the minimum ratio as shown in Eq. (6) and 5.7 corresponds to the maximum ratio as shown in Eq. (7). The search boundary for the number of values was determined through experimental testing and optimized to range between 4 and 12, ensuring effective detection of tomato objects across various conditions. The resulting optimal values were three aspect ratios [0.5, 1.0, 2] which is identical to default anchor ratios of original RetinaNet^23^, and three scales [0.2, 0.5, 0.8]. Since the TomatoPlantFactoryDataset does not provide predefined splits for training, validation, or testing, we divided the dataset into 3:1 ratio for training and validation, respectively. For evaluation, we used a 0.5 *IoU* threshold, as used in^55^ which is a common value used in agricultural object detection tasks, to calculate the *AP*. Compared to RetinaNet, which achieved AP of 85.3% for red tomatoes and 87.5% for green tomatoes, OptRetinaNet exhibited a substantial improvement, reaching AP of 90.2% for red tomatoes and 91.4% for green tomatoes. This translates to an increase of 4.9% for red tomatoes and 3.9% for green tomatoes. This performance boost demonstrates that while OptRetinaNet maintains the original aspect ratios of RetinaNet, its optimized anchor scales are key to achieving higher accuracy in object detection tasks. OptRetinaNet has also surpassed both FCOS and FSAF, as shown in Table 6 and visualized in Fig. 14. Furthermore, the detailed results in Table 6 confirm OptRetinaNet’s superior performance, as it achieved 91.6% for red tomatoes and 93.1% for green tomatoes with ResNet101, outpacing DETR and other competing methods. Additionally, Figs. 15 and 16 illustrate the AP performance and the training loss curve, respectively.

**Table 6:** The detailed results for object detection on the TomatoPlantFactoryDataset^26^ dataset are presented in terms of AP.

Model	Backbone	Red tomato (%)	Green tomato (%)
FCOS^20^	ResNet50	88.5	89.5
FSAF^19^		87.3	88.5
DETR^49^		89.4	90.0
RetinaNet^23^		85.3	87.5
OptRetinaNet		**90.2**	**91.4**
FCOS^20^	ResNet101	90.3	89.8
FSAF^19^		89.9	91.3
DETR^49^		90.4	92.6
RetinaNet^23^		89.0	89.6
OptRetinaNet		**91.6**	**93.1**
Swin-T^50^	Swin transformer	77.9	83.4
YOLOv8^41^	EfficientNet-B4	84.5	85.7

Significant values are in bold.

**Fig. 17:**
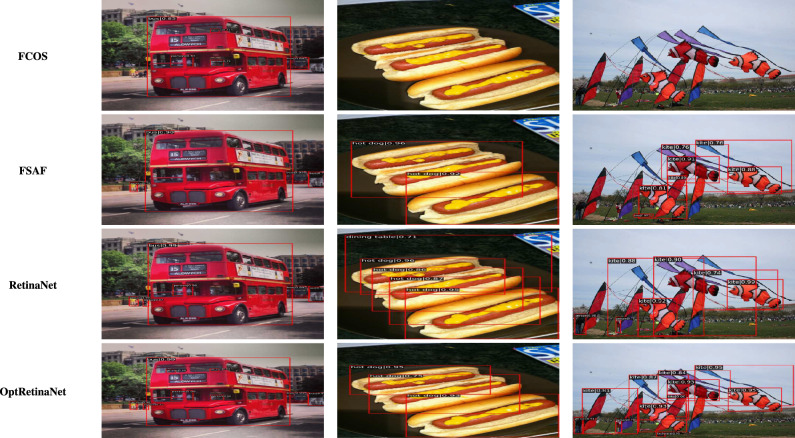
Some detection results from the models on the MS COCO 2017^27^.

**Fig. 18:**
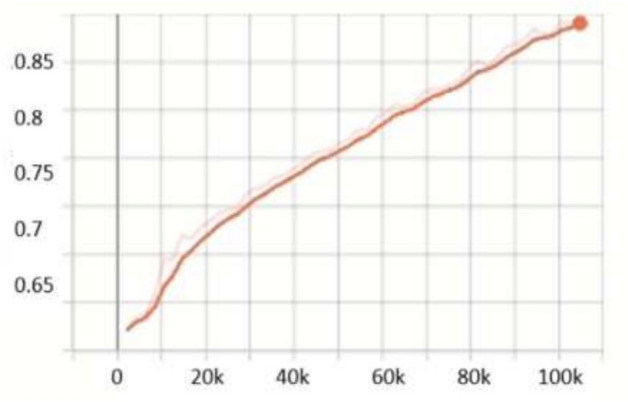
AP performance across epochs, illustrating improvements in detection accuracy over time.

**Fig. 19:**
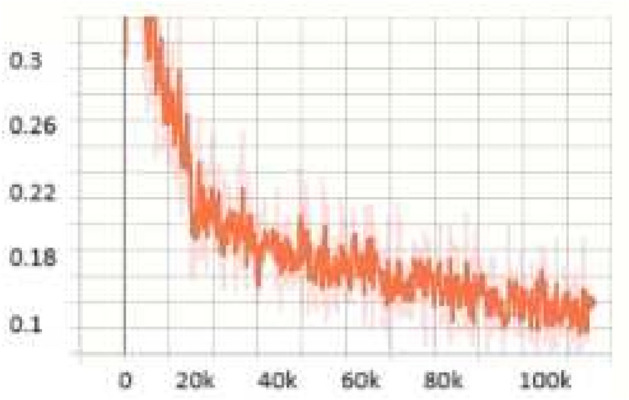
Training loss over epochs, demonstrating model convergence and stability.

For the MSCOCO 2017 dataset^27^, the scale search boundary was set to [0.06, 1.3], where 0.06 represents the minimum scale as shown in Eq. (3) and 1.3 represents the maximum scale as shown in Eq. (4). Similarly, the boundary search for aspect ratios was set to [0.15, 7.5], where 0.15 corresponds to the minimum ratio as shown in Eq. (6) and 7.5 corresponds to the maximum ratio as shown in Eq. (7). The search boundary for the number of values was determined through experimental testing and optimized to range between 3 and 12, ensuring effective detection of objects across various conditions. The resulting optimal values were five aspect ratios [0.5, 1.2, 1.5, 2.4, 3.7], and five scales [0.09, 0.13, 0.25, 0.5, 1.0]. For evaluation, we used evaluation metrics here. Compared to the baseline RetinaNet, which achieved an *AP* of 40.8% with a ResNeXt-101 backbone and 39.1% with ResNet-101, the proposed OptRetinaNet achieved significantly higher detection performance on the MS COCO 2017 dataset. OptRetinaNet attained an AP of 45.2% using ResNet-50 and 48.2% using ResNet-101, representing relative gains of 4.4% and 9.1%, respectively. These improvements extend across all evaluation metrics, including AP_50_, AP_75_, and size-specific AP scores (AP_*S*_, AP_*M*_, AP_*L*_). Notably, OptRetinaNet (ResNet-101) improved AP_*S*_ from **21.8%** in the baseline RetinaNet to **35.9%**, indicating superior performance in detecting small objects, one of the key challenges in dense object detection. Beyond improving upon RetinaNet, OptRetinaNet also outperformed several contemporary one-stage and transformer-based detection frameworks. It surpassed FCOS 44.7% AP, FSAF 37.2% AP, DETR 44.9% AP, and UP-DETR 43.1% AP, while maintaining a simpler architectural design based on standard ResNet backbones. Although state-of-the-art models such as YOLO 51.2% AP and Swin-T 50.5% AP achieved higher APs overall, OptRetinaNet demonstrated a competitive balance between performance and computational efficiency. In particular, OptRetinaNet’s AP_75_ score of **51.6%** and AP_*L*_ of **63.9%** with ResNet-101 further underscore its strength in precise localization and large-object detection. These results confirm that OptRetinaNet’s performance is primarily attributed to its DE-based optimization of anchor scales and aspect ratios, which allows the model to more effectively align with the diverse object size distribution present in complex dataset. This effectiveness is further supported by the detailed results in Table 7, with visual performance comparisons provided in Fig. 17. Additionally, Figs. 18 and 19 depict the model’s AP trends and training loss curves, emphasizing OptRetinaNet’s stable convergence and enhanced learning dynamics.

**Table 7:** The detailed results for object detection on the MSCOCO 2017^27^ dataset are presented in terms of AP.

Model	Backbone	AP (%)	AP_50_ (%)	AP_75_ (%)	AP_*S*_ (%)	AP_*M*_ (%)	AP_*L*_ (%)
FCOS^20^	ResNeXt-64x4d-101	44.7	64.1	48.4	27.6	47.5	55.6
FSAF^19^	ResNet50	37.2	57.2	39.4	21.0	41.2	49.7
DETR^49^	ResNet50	44.9	64.7	47.7	23.7	49.5	62.3
RetinaNet^23^	ResNeXt-101	40.8	61.1	44.1	24.1	44.2	52.2
OptRetinaNet	ResNet50	45.2	64.9	51.4	32.5	53.6	60.2
RetinaNet^23^	ResNet101	39.1	59.1	42.3	21.8	42.7	50.2
OptRetinaNet	ResNet101	48.2	64.4	51.6	**35.9**	55.8	63.9
Swin-T^50^	Swin transformer	50.5	69.3	54.9	–	–	–
YOLO^41^	EfficientNet-B4	**51.2**	**69.7**	**55.5**	35.2	**56.0**	**66.7**
CutLER^52^	ResNet101	12.3	21.9	11.8	3.7	12.7	29.6
UP-DETR^51^	ResNet101	43.1	63.4	46.0	21.6	46.8	62.4

Significant values are in bold.

## Dataset availability

This study utilized publicly available datasets. The datasets used in this study can be accessed as follows:

- *KITTI dataset:* available at https://www.cvlibs.net/datasets/kitti/.
- *UFFD dataset:* available at https://ufdd.info/.
- *TomatoPlantFactoryDataset:* available at https://data.mendeley.com/datasets/8h3s6jkyff/1.
- *MS COCO 2017:* available at https://cocodataset.org/#download.

All datasets are openly accessible and were used in accordance with their respective terms of use. No additional ethical approval was required for their use.

## Limitations and future work

### Limitations

The proposed approach may face challenges in detecting highly occluded objects, as anchor-based methods inherently rely on visible object features for accurate localization. In scenarios where objects are partially hidden or heavily obstructed, the optimization process may fail to generate effective anchors, leading to missed detections or reduced precision. Additionally, the iterative nature of differential evolution introduces a higher computational cost, particularly when applied to large-scale datasets with a vast number of object instances. This increased complexity may pose challenges in applications with limited computational resources, requiring the development of efficient acceleration techniques to enhance processing speed while maintaining detection accuracy.

### Future work

To further improve the adaptability and efficiency of the proposed method, several future directions can be explored: *Multi-Task Learning:* Extending the approach to incorporate multi-task learning, such as jointly optimizing anchor configurations and feature representations, could enhance model generalization across different datasets. *Self-Supervised Optimization:* Exploring self-supervised learning for anchor adaptation could enable the model to refine anchor parameters without requiring large amounts of annotated data. *Temporal Consistency for Video-Based Detection:* Extending the method to video object detection by optimizing anchor configurations across frames could improve tracking stability in real-time applications. By addressing these limitations and expanding the methodology in future work, the proposed optimization strategy can be further refined to enhance robustness, reduce computational complexity, and improve detection accuracy in various real-world applications.

## Supplementary Information

Supplementary Information.

## Data Availability

The datasets used in this study are publicly available as follows: Dataset 1: KITTI dataset, accessible at https://www.cvlibs.net/datasets/kitti/ Dataset 2: UFFD dataset, accessible at https://ufdd.info/ Dataset 3: TomatoPlantFactoryDataset , accessible at https://data.mendeley.com/datasets/8h3s6jkyff/1 Dataset 4: MS COCO 2017 , accessible at https://cocodataset.org/#download All datasets are openly accessible and were used in accordance with their respective terms of use.
